# Mediolateral Joint Balance Assessment Is Reproducible Between Surgeons, Under No Standardized Varus–Valgus Stress, During Robot-Assisted Total Knee Arthroplasty

**DOI:** 10.3390/jcm15207926

**Published:** 2026-10-14

**Authors:** Giovanni Grillo, Alexandre Coelho Leal, David Martí, Marçal Pineda Tortajada, Sergi Gil-Gonzalez, Juan Manuel Peñalver Matamoros, Xavier Pelfort Lopéz

**Affiliations:** 1 Knee Unit, Department of Orthopaedics and Traumatology, Parc Taulí Hospital Universitari, Institut d’Investigació I Innovació Parc Taulí (I3PT-CERCA), Universitat Autònoma Barcelona (UAB), 08208 Sabadell, Spain; acoelho@tauli.cat (A.C.L.); dmarti@tauli.cat (D.M.); sgilg@tauli.cat (S.G.-G.); jpenalver@tauli.cat (J.M.P.M.); xpelfort@tauli.cat (X.P.L.); 2 3D Surgical Planning Lab, Parc Taulí Hospital Universitari, Institut d’Investigació I Innovació Parc Taulí (I3PT-CERCA), Universitat Autònoma de Barcelona (UAB), 08208 Sabadell, Spain; 3 Medicine and Surgery Faculty, Universitat Autonoma de Barcelona, 08193 Bellaterra, Spain; dr.pinedatortajada@gmail.com

**Keywords:** ROSA knee system, robotically assisted system, total knee arthroplasty

## Abstract

**Objective:** To evaluate inter-surgeon variability in mediolateral joint balance assessment during robotic-assisted total knee arthroplasty (TKA) using the robotic surgical assistant (ROSA) Knee System, and to determine whether such variability influences the surgical planning generated by this system. **Methods:** An observational study was conducted including 101 patients undergoing primary robotic-assisted TKA. Two experienced surgeons independently assessed intraoperative mediolateral laxity by manually applying varus–valgus stress across six predefined flexion angles (0°, 30°, 45°, 60°, 90°, and 120°). Measurements of medial and lateral gaps (mm) and varus–valgus alignment (°) were recorded using the ROSA Knee System. Inter-observer differences were analysed using the Wilcoxon signed-rank test, and reliability was assessed using the intraclass correlation coefficient (ICC). The impact of inter-surgeon variability on surgical planning, including bone resection and compartmental space measurement parameters, was also evaluated. **Results:** No statistically significant inter-observer differences were observed in mediolateral gap measurements or varus–valgus alignment across all flexion angles (*p* > 0.05). Inter-observer reliability for varus–valgus measurements was good to excellent in extension and early flexion (ICC 0.86–0.91), decreased to moderate at 90° (ICC 0.68–0.74) and was poor for varus assessment at 120° (ICC 0.46, 95% CI 0.29–0.60). For gap measurements, reliability was excellent for the medial gap and good for the lateral gap in extension (ICC 0.93 and 0.89, respectively), and moderate for both gaps at 90° (ICC 0.71–0.74). No significant inter-observer differences were found in cut or space measurements, with excellent agreement for cut parameters (ICC > 0.95). **Conclusions:** Inter-surgeon assessment of mediolateral joint balance during robot-assisted TKA showed good-to-excellent reproducibility in extension and early flexion, with reduced reproducibility in deep flexion. Statistically significant inter-observer differences were not observed in the resulting cut and space measurements.

## 1. Introduction

Total knee arthroplasty (TKA) is a highly successful procedure for the treatment of end-stage knee osteoarthritis [1]. Achieving optimal soft-tissue balance remains a critical determinant of postoperative function, patient satisfaction, and implant longevity. Despite advances in implant design and surgical techniques, residual imbalance in the mediolateral compartments has been associated with pain, instability, and early failure. Consequently, accurate and reproducible assessment of joint laxity throughout the range of motion is essential for achieving a well-balanced knee [2,3]. Intraoperative varus–valgus assessment provides a dynamic evaluation of mediolateral ligament laxity and is therefore an important component of soft-tissue balancing during TKA, helping to identify residual asymmetry or instability and contributing to intraoperative decision-making [2,3].

Robotic-assisted TKA has been increasingly integrated with the aim of improving the precision and reproducibility of implant positioning and intraoperative decision-making [4]. Modern robotic platforms allow real-time assessment of joint gaps and limb alignment across multiple degrees of flexion, enabling a more comprehensive and dynamic evaluation compared with conventional techniques. These systems integrate intraoperative data to guide bone resections and implant positioning in the coronal, sagittal, and axial planes, with the potential to standardize surgical execution and reduce technical variability [5,6].

Nevertheless, mediolateral gap assessment during robotic-assisted TKA remains dependent on manual application of varus–valgus stress by the surgeon. The magnitude and consistency of the applied force are not standardized, introducing a potential source of variability even when robotic systems are used. While previous studies have shown that surgeon experience influences the reproducibility of gap assessment, the extent to which inter-surgeon variability persists in the setting of robotic assistance, and whether it affects surgical planning, remains unclear [7,8,9,10,11,12].

The purpose of this study was to evaluate inter-surgeon variability in mediolateral joint balance throughout the range of motion during robotic-assisted TKA using the ROSA Knee System (Zimmer Biomet, Warsaw, IN, USA) and to determine whether such variability influences the surgical planning generated by the robotic system.

## 2. Materials and Methods

An observational study was conducted to evaluate the reliability of intraoperative knee joint balancing assessment using the ROSA Knee System (Zimmer Biomet, Warsaw, IN, USA).

Sample size estimation for the inter-observer reliability study was performed using GRANMO software (version 8.0). Based on a two-rater ICC model, assuming an expected ICC of 0.85, a null-hypothesis ICC of 0.75, a two-sided α level of 0.05, and 80% statistical power, the minimum required sample size was 99 knees. No adjustment for dropouts was applied because all measurements were collected during the index surgical procedure. No loss to follow-up was anticipated. The study protocol was approved by the institutional review board with protocol number 2025/5039, all procedures were conducted in accordance with the Declaration of Helsinki, and all patients signed the informed consent. The study included patients treated both before and after the approval date. Intraoperative measurements were part of routine clinical practice and were not introduced for research purposes. Patients treated before approval were included retrospectively, whereas subsequent patients were included prospectively.

Patients undergoing primary robotic-assisted TKA between September 2023 and December 2025 were included in the study following inclusion and exclusion criteria.

Inclusion criteria were as follows: age ≥ 60 years; diagnosis of primary knee osteoarthritis classified as grade III or IV according to the Ahlbäck classification; presence of symptomatic knee pain requiring surgical treatment; eligibility for elective primary total knee arthroplasty; American Society of Anesthesiologists (ASA) physical status I–IV; and provision of written informed consent.

Exclusion criteria included: secondary osteoarthritis; previous knee surgery or prior knee implants (e.g., plates, screws, or other fixation devices); severe coronal deformity (valgus or varus > 15°); flexion contracture > 20°; history of previous knee fracture; congenital or acquired deformities affecting the knee joint; contraindications to total knee arthroplasty; and inability or refusal to provide informed consent.

All patients underwent standardized preoperative clinical and radiographic evaluation, including anteroposterior and lateral knee radiographs, Rosenberg and Schuss views, and full-length weight-bearing long-leg radiographs [13]. These were used to confirm the indication for surgery and for preoperative planning.

All procedures were performed using the ROSA Knee System (Zimmer Biomet, Warsaw, IN, USA). Following standard surgical exposure and osteophyte removal, femoral and tibial landmarks were acquired to establish the anatomical reference framework for robotic-assisted planning [14].

After landmark acquisition, mediolateral knee laxity was independently assessed by the surgeons. Varus and valgus stresses were manually applied and a single measurement was recorded for each condition, without repeated measurements. For each assessment, the knee was evaluated sequentially from 0° to 120° of flexion at six predefined angles (0°, 30°, 45°, 60°, 90°, and 120°). Varus stress was applied first, followed by valgus stress. No device was used to standardize or quantify the magnitude of the applied varus–valgus stress; therefore, neither the magnitude of the applied force nor a force-defined endpoint was standardized. The endpoint was determined by each surgeon according to their usual intraoperative assessment of ligamentous balance, reflecting routine clinical practice.

Measurements were obtained at six predefined flexion angles: 0° (full extension), 30°, 45°, 60°, 90°, and 120°. The ROSA Knee System automatically provided medial and lateral compartment gaps (mm, at only 0° and 90°) and varus–valgus laxity (°).

All measurements were recorded using the system’s screenshot function for subsequent analysis.

Intraoperative joint balancing assessments were independently performed by two experienced orthopedic surgeons, each blinded to the other’s measurements. During each assessment, only the surgeon performing the maneuver was permitted to visualize the real-time ROSA display. The other surgeon remained blinded to the maneuver and measurements by turning away from the operative field and display during the assessment.

Because no universally accepted definition of an expert surgeon in robotic-assisted TKA exists [15], surgeon experience was defined a priori using procedural volume and platform-specific experience. In the present study, both surgeons were experienced knee arthroplasty surgeons who had used the ROSA Knee System for more than 5 years and had performed more than 100 ROSA-assisted TKAs before study initiation. Therefore, they were considered as experienced robotic TKA surgeons for the purposes of this inter-observer reliability study.

The surgical plan generated by the ROSA Knee System (Zimmer Biomet, Warsaw, IN, USA) was recorded for each procedure.

The final implant positioning and surgical plan were based on the evaluation of the primary surgeon.

The dependent variables were mediolateral knee varus–valgus, expressed in degrees (°), measured at the predefined flexion angles; medio and lateral gaps expressed in millimeter (mm) and; planning cut expressed in millimeter (mm).

Also, femoral size implant and femoral notching were collected.

The surgeon performing the intraoperative assessment was treated as a two-level categorical variable (Surgeon A and Surgeon B).

All data were analyzed. The distribution of paired differences between measurements obtained by the two surgeons was assessed using the Shapiro–Wilk test. As paired differences were not normally distributed for most comparisons, inter-observer comparisons were performed using the Wilcoxon signed-rank test. Continuous variables are presented as mean ± standard deviation (SD) to provide descriptive estimates of measurement magnitude and dispersion. Categorical variables are reported as counts and percentages. A *p*-value > 0.05 was interpreted as absence of evidence of a systematic paired difference and not as evidence of equivalence or agreement.

Inter-observer reliability for continuous measurements was evaluated using the intraclass correlation coefficient (ICC), based on a two-way random-effects model with absolute agreement for single measurements [ICC (2,1)]. ICC estimates are reported with their 95% confidence intervals. ICC values were interpreted as poor (<0.50), moderate (0.50–0.75), good (0.75–0.90), or excellent (>0.90) [16]. Agreement for categorical variables was assessed using Cohen’s kappa coefficient.

Agreement between surgeons was further evaluated using Bland–Altman analysis. The distribution of paired differences was assessed using the Shapiro–Wilk test, and potential heteroscedasticity was examined by evaluating the relationship between the magnitude of the measurements and the absolute inter-observer differences. Because paired differences showed significant departures from normality for the varus–valgus and gap measurements, a non-parametric Bland–Altman approach was used. Bias was expressed as the median paired difference (Surgeon A − Surgeon B), and the lower and upper limits of agreement (LoA) were estimated as the 2.5th and 97.5th percentiles of the paired differences, respectively. Ninety-five percent confidence intervals for the median bias and both limits of agreement were estimated using bootstrap resampling.

All statistical analyses were performed using R statistical software (version 4.5.3). ICC was computed using the psych package, Bland–Altman plots using the BlandAltmanLeh package, and tables were generated using the kable package. A two-sided *p*-value < 0.05 was considered statistically significant.

## 3. Results

A total of 101 patients who met the predefined inclusion criteria were included in the study, corresponding to 101 knees. Complete varus–valgus and medial and lateral gap measurements were available for all included knees. The study population consisted of 68 females (67.3%) and 33 males (32.7%), with a mean age of 72.2 ± 7.9 years. The mean body mass index was 31.6 ± 5.1 kg/m^2^. The distribution of surgical roles between the two surgeons, was balanced, with Surgeon A acting as first surgeon in 44 cases (43.6%) and second surgeon in 57 cases (56.4%), and Surgeon B acting as first surgeon in 57 cases (56.4%) and second surgeon in 44 cases (43.6%).

### 3.1. Inter-Observer Comparison of Varus–Valgus Alignment

No statistically significant differences were observed between the first and second surgeon, using the Wilcoxon signed-rank test for varus or valgus alignment at any flexion angle, as it shown in Table 1. Agreement and reliability were subsequently evaluated using Bland–Altman analysis and ICC, respectively.

Similarly, no significant inter-observer differences were detected at 30°, 45°, 60°, 90° and 120° of flexion, with median values remaining comparable across all angles.

#### 3.1.1. Inter-Observer Reliability of Varus–Valgus Measurements

Inter-observer reliability analysis demonstrated good to excellent agreement for varus and valgus measurements across most flexion angles (Table 2). ICC values ranged from 0.86 to 0.91 in extension and early flexion (0–30°), indicating good to excellent reliability. At mid-flexion angles (45–60°), ICC values remained within the good range.

A reduction in ICC values was observed at 90° and 120° of flexion, with moderate agreement at 90° and poor agreement for varus measurements at 120°. This reduction occurred despite comparable mean values between surgeons and likely reflects increased measurement variability in deep flexion rather than systematic inter-observer differences.

Bland–Altman analysis confirmed the absence of systematic inter-observer bias for both varus and valgus alignment (Figure 1 and Figure 2).

#### 3.1.2. Inter-Observer Comparison of Medial and Lateral Gaps

Medial and lateral gap measurements showed no statistically significant differences between surgeons in either extension or flexion, as it shown in Table 3.

Inter-observer reliability for gap measurements was excellent for the medial gap (ICC 0.93, 95% CI 0.90–0.95) and good for the lateral gap (ICC 0.89, 95% CI 0.85–0.93) at 0°. At 90°, reliability was moderate for both the medial gap (ICC 0.71, 95% CI 0.60–0.80) and lateral gap (ICC 0.74, 95% CI 0.64–0.82).

Non-parametric Bland–Altman analysis showed minimal systematic inter-observer bias, with a median paired difference of 0° across varus and valgus assessments. However, the limits of agreement widened with increasing knee flexion, particularly in deep flexion. For varus assessment, the limits of agreement widened from −3.0° (95% CI −4.5 to −2.0) and 3.0° (95% CI 2.0 to 10.5) at 0° to −10.5° (95% CI −17.0 to −6.5) and 10.0° (95% CI 7.0 to 14.0) at 120°. At 120°, valgus assessment showed limits of agreement of −8.5° (95% CI −13.0 to −5.5) and 7.0° (95% CI 4.5 to 9.0) (Table 4). Gap measurements similarly showed a median bias of 0 mm, with wider limits of agreement at 90° than at 0°. The corresponding non-parametric Bland–Altman plots are shown in Figure 1 and Figure 2.

#### 3.1.3. Inter-Observer Comparison of Cut and Space Measurements

Cut and space measurements in both extension and flexion did not differ significantly between surgeons (Table 5). Medial and lateral cut values in extension and flexion demonstrated comparable mean and median values, with all comparisons showing non-significant differences.

Similarly, medial and lateral space measurements showed no statistically significant inter-observer differences, although a trend toward greater variability was observed for medial space measurements, particularly in flexion.

Reliability analysis revealed excellent agreement for cut measurements in both extension and flexion, with ICC values exceeding 0.95. Space measurements demonstrated good to excellent reliability in extension, while moderate reliability was observed for medial space measurements in flexion (ICC = 0.56), consistent with increased soft-tissue variability.

#### 3.1.4. Inter-Observer Agreement for Categorical Variables

Perfect inter-observer agreement was observed for femoral component size selection (weighted Cohen’s κ = 1.00) and notching assessment (Cohen’s κ = 1.00).

## 4. Discussion

The primary finding of the present study is that inter-surgeon assessment of mediolateral joint balance showed good-to-excellent reproducibility in extension and early flexion, with reduced reproducibility at higher degrees of flexion. This reduction was most pronounced for varus assessment at 120°, which showed poor reliability (ICC 0.46, 95% CI 0.29–0.60). Consistent with these findings, Bland–Altman analysis showed wider limits of agreement in deep flexion, indicating greater inter-observer variability despite minimal systematic bias. These results indicate that intraoperative joint-balance assessment showed generally consistent measurements between experienced operators at the flexion angles relevant to the surgical planning evaluated in this study using the ROSA system, despite the manual application of non-standardized varus–valgus stress.

Our findings should be interpreted in the context of previous studies evaluating the reproducibility of manual ligament assessment during robotic-assisted TKA. Yee et al. assessed inter- and intra-rater reliability in 24 knees involving surgeons with different levels of experience [10]. Corbett et al. evaluated manual stress assessment in a cohort of 123 patients across six surgeon–assistant pairs and also compared manual assessment with instrumented distraction [11]. More recently, Everaert et al. investigated manual soft-tissue assessment and its influence on surgical planning using the same ROSA robotic platform, although their study included 17 patients and primarily compared a high-volume surgeon with a low-volume resident [12]. Similarly, Sohmiya et al. reported that surgeon experience may influence the reproducibility of gap assessment under manual stress, with differences observed between experienced and inexperienced surgeons [17].

The present study extends these findings by evaluating 101 enrolled TKAs assessed by two surgeons both experienced with the same robotic platform and by examining varus–valgus laxity at six predefined flexion angles from 0° to 120°. This broader assessment across the range of motion allowed us to characterize the progressive reduction in inter-surgeon reliability with increasing flexion, particularly for varus assessment in deep flexion. Thus, rather than establishing the reproducibility of manual assessment per se, our findings provide additional information on how inter-surgeon reproducibility varies throughout the range of motion when assessments are performed by experienced robotic TKA surgeons without force-standardized varus–valgus stress.

A key methodological aspect of this study is that the magnitude of varus–valgus stress applied during intraoperative assessment was neither standardized nor quantified. While this may be considered a limitation from a purely methodological standpoint, it reflects current clinical practice, in which ligament balancing is typically performed manually without the use of force-controlled devices.

This approach was intentional. The aim of the present study was to evaluate inter-surgeon variability under routine conditions, in which the applied varus–valgus force was not standardized or quantified. Introducing force standardization would have addressed a different experimental question and would not have reflected the assessment protocol evaluated in the present study.

Although the magnitude of the applied force was not measured and may have differed between surgeons, the present study was designed to reproduce routine intraoperative practice without force quantification. Under these conditions, the resulting measurements showed generally good inter-surgeon reproducibility in extension and early flexion, whereas reproducibility decreased in deep flexion, particularly for varus assessment at 120°. Future studies directly measuring or controlling the applied force could determine the extent to which force variability contributes to these differences.

From a clinical standpoint, these results indicate that, among the two experienced surgeons evaluated in this study, manual varus–valgus stress application without force standardization or quantification resulted in generally reproducible joint-balance measurements, particularly in extension and early flexion. Importantly, this finding relates to the reproducibility of the resulting measurements and should not be interpreted as evidence that the surgeons applied equivalent forces.

Previous studies have explored the use of instrumented tensioning devices to quantify ligament forces and improve the reproducibility of gap assessment during total knee arthroplasty [17,18,19]. A recent meta-analysis by R. Becker [20] demonstrated that distraction forces in TKA can be quantified, reporting mean force values at 0° and 90°, providing reference values, and suggesting consistency in the forces applied across different experimental models. However, it remains unclear whether standardized distraction forces are appropriate for all patients or whether a more individualized approach to ligament balancing should be considered [4]. In the present study, joint-balance assessment and the resulting surgical planning measurements were obtained using a ROSA-assisted workflow without dedicated force-measuring devices. The reproducibility observed under routine manual assessment without force quantification provides a basis for future comparative studies evaluating whether force-controlled assessment can further improve inter-surgeon reproducibility, particularly in deep flexion where greater variability was observed. Instrumented tensioning devices allow quantification and standardization of the applied distraction force, an aspect that was intentionally not evaluated in the present study. Therefore, our findings should not be interpreted as evidence against the potential value of force-controlled devices. Rather, they provide information on the reproducibility achievable between experienced surgeons when joint balance is assessed manually without force quantification. Direct comparative studies would be required to determine whether force-controlled devices further improve reproducibility.

A secondary finding of this study is the reduction in inter-observer reliability at higher degrees of flexion. Despite comparable mean values, agreement decreased at 90° and, more markedly, at 120° of flexion. This was evident for varus assessment at 120°, for which the point estimate indicated poor reliability (ICC 0.46, 95% CI 0.29–0.60). Although the point estimate falls within the predefined poor reliability category, the confidence interval crosses the threshold for moderate reliability, indicating uncertainty around this categorical classification. Importantly, this reduced reliability occurred in the absence of a systematic difference between surgeons, as mean varus measurements at 120° were similar. Thus, the lower ICC should not be interpreted as systematic inter-surgeon bias, but rather as reduced reproducibility of individual measurements in deep flexion. The reason for this reduction cannot be established from the present study. However, deep-flexion assessment may be more sensitive to differences in limb positioning, joint geometry, soft-tissue behavior, and manual stress application. Because the magnitude of the applied varus–valgus force was neither standardized nor quantified, the contribution of force variability to the lower reliability observed at 120° cannot be determined. These findings therefore suggest that measurements obtained in deep flexion, particularly varus assessment at 120°, should be interpreted with greater caution than measurements obtained in extension and early flexion.

This finding likely reflects the greater complexity of soft-tissue behavior in deep flexion, where ligament tension, joint geometry, and force application become more difficult to standardize [21].

It should also be noted that, within the ROSA workflow used in this study, the measurements used for final surgical planning are obtained at 0° and 90° of flexion. Therefore, the reduced reproducibility observed at 120°, particularly for varus assessment, should be interpreted as a limitation of manual joint-balance assessment in deep flexion rather than as a direct source of variability in the final surgical planning evaluated in this study.

The non-parametric Bland–Altman analysis further supports these findings, demonstrating minimal systematic bias between surgeons, with median paired differences close to zero for both alignment and gap measurements. However, the limits of agreement widened with increasing flexion, indicating greater inter-observer dispersion in deep flexion despite minimal systematic bias.

Importantly, the absence of statistically significant differences between surgeons should not be interpreted as the absence of variability. Rather, the observed variability was predominantly non-directional, without evidence of systematic bias between operators. In this cohort, this variability was not accompanied by statistically significant inter-observer differences in the resulting cut and space measurements.

Regarding the next objective of the study, no significant inter-observer differences were observed in cut or space measurements generated by the ROSA Knee System. Statistically significant inter-observer differences were not observed in the resulting cut and space measurements. These results are in line with the literature because minor differences in ligamentous evaluation do not appear to influence the final surgical plan [4,19].

However, these findings should not be interpreted as demonstrating equivalence between surgeons or as excluding potentially relevant differences at the individual-patient level.

A study by Figueroa et al. [21] focused on the reliability in reproducing the planned cuts with a robotic-arm system that incorporates robotic placement of the cutting jigs. In their study, they reported high concordance between planned and achieved resections using a robotic system, supporting the reliability of robotic planning and execution. This suggests that the robotic planning algorithm is robust to small variations in intraoperative measurements, and confirms it is possible to perform accurate bone planning cuts and to achieve the planned angles and resections [4].

From a clinical standpoint, the consistency observed in cut and space measurements suggests that the inter-surgeon variability observed in manual joint-balance assessment was not accompanied by substantial differences in the resulting surgical planning measurements in this cohort. These findings suggest that, within the ROSA-assisted workflow evaluated in this study, some inter-surgeon variability in manual joint-balance assessment may occur without corresponding statistically significant differences in the resulting cut and space measurements.

This study has some limitations. First, only two experienced surgeons were included, which may limit the generalizability of the findings. Second, the study did not include clinical or radiological outcomes, and therefore no conclusions can be drawn regarding the impact of inter-observer variability on postoperative results. Third, although the role of first and second surgeon was relatively balanced between the two observers, the order of assessment was not randomized. Therefore, a potential order effect, including tissue preconditioning or carry-over from the preceding manual varus–valgus stress assessment, cannot be completely excluded. Finally, the relatively low ICC observed for varus assessment at 120° (ICC 0.46, 95% CI 0.29–0.60) represents a limitation when interpreting reproducibility in deep flexion, particularly given the uncertainty reflected by its confidence interval.

## 5. Conclusions

Inter-surgeon assessment of mediolateral joint balance during robot-assisted TKA showed good-to-excellent reproducibility in extension and early flexion, with reduced reproducibility in deep flexion. Inter-surgeon variability was not accompanied by statistically significant differences in the resulting cut and space measurements. Further studies specifically designed to assess equivalence and clinically relevant individual differences are warranted to determine their potential impact on surgical planning.

Declaration of conflicting interests: The authors declare no potential conflicts of interest with respect to the research, authorship, and/or publication of this article.

## Figures and Tables

**Figure 1:**
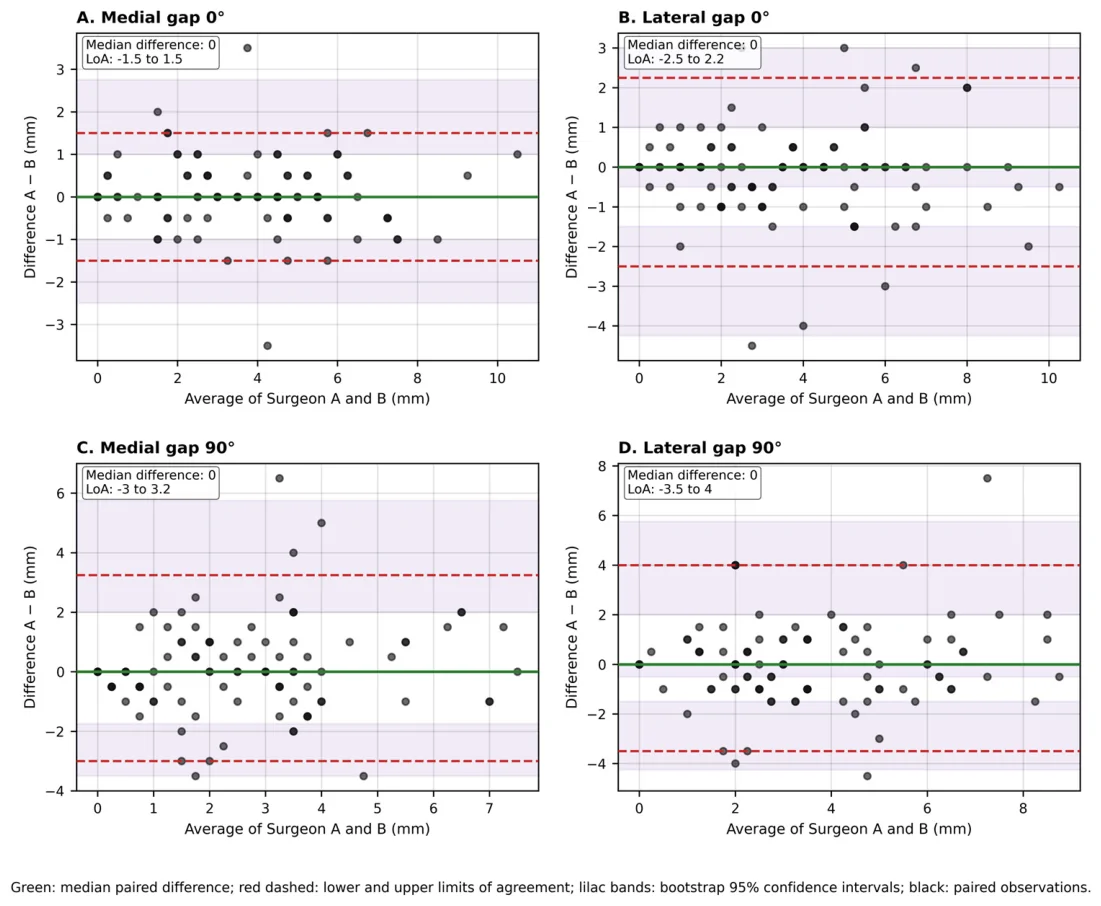
Non-parametric Bland–Altman plots demonstrating agreement between Surgeon A and Surgeon B for varus and valgus alignment. The solid horizontal line represents the median paired difference (Surgeon A − Surgeon B), while the dashed lines represent the non-parametric limits of agreement defined by the 2.5th and 97.5th percentiles of the paired differences.

**Figure 2:**
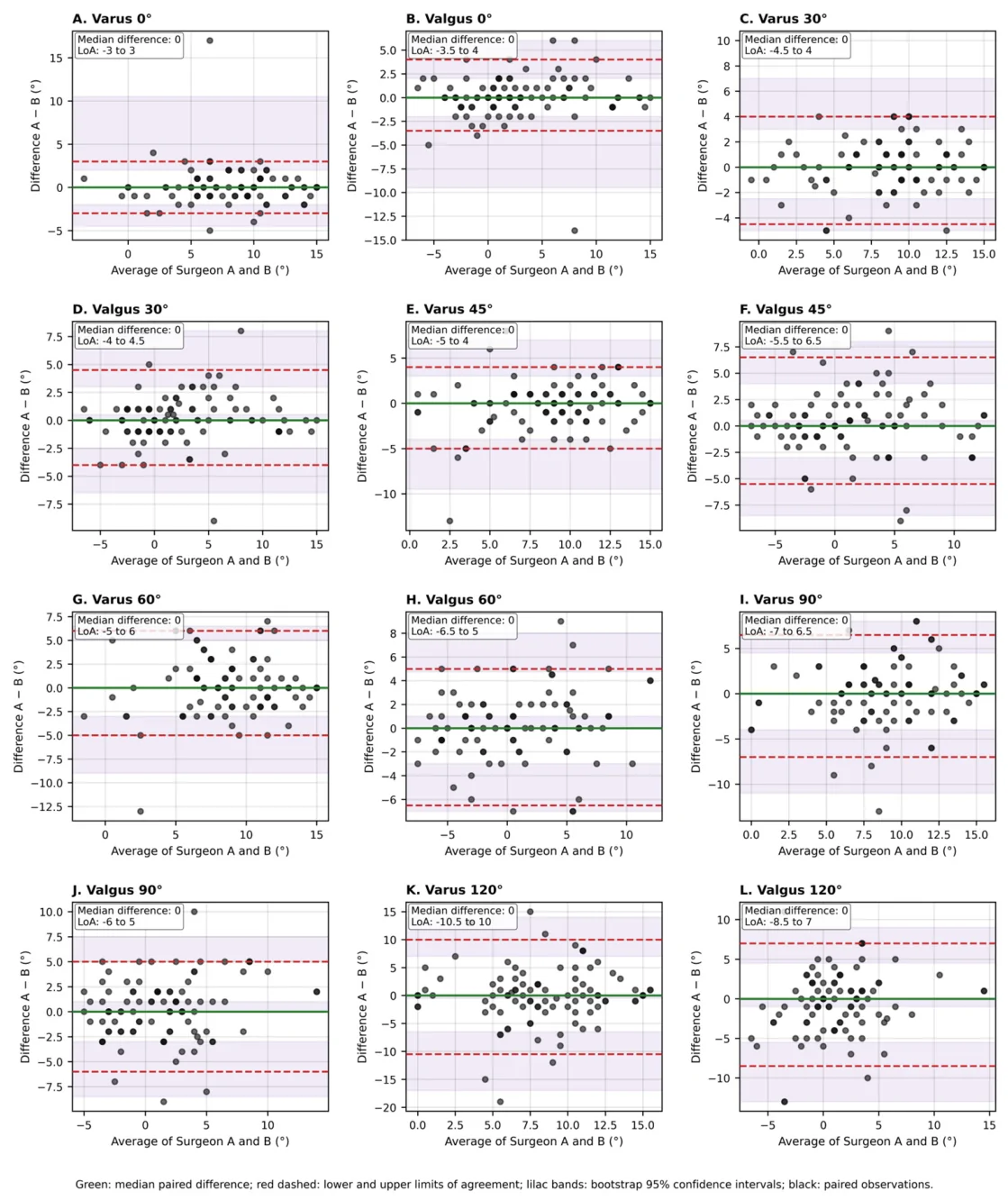
Non-parametric Bland–Altman plots demonstrating agreement between Surgeon A and Surgeon B for varus and valgus alignment and medial and lateral gap measurements. The solid horizontal line represents the median paired difference (Surgeon A − Surgeon B), while the dashed lines represent the non-parametric limits of agreement defined by the 2.5th and 97.5th percentiles of the paired differences.

**Table 1:** Inter-observer comparison of varus–valgus alignment at different flexion angles.

Flexion Angle	Measurement	Surgeon A (Mean ± SD)	(Median ± IQR A)	Surgeon B (Mean ± SD)	(Median ± IQR B)	*p*-Value
0°	varus	8.6 ± 4.2°	9 [6–12]	8.5 ± 4.3°	9 [6–11]	0.885
0°	valgus	3.0 ± 5.3°	2 [−1–7]	2.9 ± 5.2°	2 [0–6]	0.404
30°	varus	9.2 ± 4.0°	10 [7–12]	9.0 ± 4.0°	9 [6–12]	0.487
30°	valgus	2.6 ± 5.3°	2 [−1–6]	2.5 ± 5.2°	1 [−1–5]	0.422
45°	varus	9.1 ± 4.3°	9 [7–12]	9.3 ± 3.8°	9 [6–12]	0.437
45°	valgus	1.6 ± 5.1°	1 [−3–5]	1.4 ± 5.1°	0 [−3–5]	0.362
60°	varus	9.2 ± 4.1°	9 [7–12]	9.1 ± 4.0°	9 [7–12]	0.783
60°	valgus	0.8 ± 5.1°	0 [−3–4]	0.6 ± 4.6°	0 [−3–4]	0.352
90°	varus	9.3 ± 4.3°	9 [6–12]	9.4 ± 3.9°	9 [7–12]	0.835
90°	valgus	1.5 ± 4.4°	1 [−2–3]	1.2 ± 3.9°	1 [−2–3]	0.325
120°	varus	8.5 ± 4.9°	9 [6–12]	8.7 ± 4.5°	9 [6–12]	0.992
120°	valgus	0.9 ± 4.3°	0 [−1–3]	1.4 ± 3.6°	1 [0–3]	0.191

**Table 2:** Inter-observer reliability analysis (ICC).

Variable	ICC (2,1)	95% CI	Interpretation
Varus 0°	0.86	0.80–0.90	Good
Valgus 0°	0.91	0.87–0.94	Excellent
Varus 30°	0.86	0.80–0.91	Good
Valgus 30°	0.91	0.87–0.94	Excellent
Varus 45°	0.79	0.70–0.85	Good
Valgus 45°	0.85	0.78–0.89	Good
Varus 60°	0.73	0.62–0.81	Moderate
Valgus 60°	0.83	0.76–0.88	Good
Varus 90°	0.68	0.56–0.77	Moderate
Valgus 90°	0.74	0.63–0.81	Moderate
Varus 120°	0.46	0.29–0.60	Poor
Valgus 120°	0.54	0.39–0.67	Moderate
Medial gap 0°	0.93	0.90–0.95	Excellent
Lateral gap 0°	0.89	0.85–0.93	Good
Medial gap 90°	0.71	0.60–0.80	Moderate
Lateral gap 90°	0.74	0.64–0.82	Moderate

**Table 3:** Inter-observer comparison of medial and lateral gaps.

Gap Measurement (mm)	Surgeon A (Mean ± SD)	(Median ± IQR A)	Surgeon B (Mean ± SD)	(Median ± IQR B)	*p*-Value
Medial gap 0°	3.71 ± 2.24	3.5 [2.5–5.0]	3.65 ± 2.31	3.5 [2.0–5.0]	0.468
Lateral gap 0°	3.56 ± 2.48	3 [1.5–5.0]	3.75 ± 2.52	3.5 [1.5–5.5]	0.065
Medial gap 90°	2.55 ± 2.12	2.5 [0.5–3.5]	2.45 ± 1.93	2 [1.0–3.5]	0.632
Lateral gap 90°	3.45 ± 2.42	3 [2.0–5.0]	3.41 ± 2.26	3 [2.0–5.0]	0.891

**Table 4:** Non-parametric Bland–Altman analysis of inter-observer agreement.

Measurement	n	Median Bias (95% CI)	Lower LoA (95% CI)	Upper LoA (95% CI)
Varus 0°	101	0.0 (0.0 to 0.0)	−3.0 (−4.5 to −2.0)	3.0 (2.0 to 10.5)
Valgus 0°	101	0.0 (0.0 to 0.0)	−3.5 (−9.5 to −2.0)	4.0 (2.0 to 6.0)
Varus 30°	101	0.0 (0.0 to 0.0)	−4.5 (−5.0 to −2.5)	4.0 (3.0 to 7.0)
Valgus 30°	101	0.0 (0.0 to 0.5)	−4.0 (−6.5 to −2.5)	4.5 (3.0 to 8.0)
Varus 45°	101	0.0 (0.0 to 0.0)	−5.0 (−9.5 to −4.0)	4.0 (3.0 to 7.0)
Valgus 45°	101	0.0 (0.0 to 0.5)	−5.5 (−8.5 to −3.0)	6.5 (4.0 to 8.0)
Varus 60°	101	0.0 (0.0 to 0.0)	−5.0 (−9.0 to −3.0)	6.0 (5.0 to 6.5)
Valgus 60°	101	0.0 (0.0 to 1.0)	−6.5 (−7.0 to −3.0)	5.0 (4.8 to 8.0)
Varus 90°	101	0.0 (0.0 to 0.0)	−7.0 (−11.0 to −4.0)	6.5 (4.5 to 8.0)
Valgus 90°	101	0.0 (0.0 to 1.0)	−6.0 (−8.5 to −3.0)	5.0 (5.0 to 7.5)
Varus 120°	101	0.0 (−0.5 to 0.0)	−10.5 (−17.0 to −6.5)	10.0 (7.0 to 14.0)
Valgus 120°	101	0.0 (−1.0 to 0.0)	−8.5 (−13.0 to −5.5)	7.0 (4.5 to 9.0)
Medial gap 0°	101	0.0 (0.0 to 0.0)	−1.5 (−2.5 to −1.0)	1.5 (1.0 to 2.8)
Lateral gap 0°	101	0.0 (−0.5 to 0.0)	−2.5 (−4.2 to −1.5)	2.2 (1.0 to 3.0)
Medial gap 90°	101	0.0 (0.0 to 0.0)	−3.0 (−3.5 to −1.8)	3.2 (2.0 to 5.8)
Lateral gap 90°	101	0.0 (−0.5 to 0.0)	−3.5 (−4.2 to −1.5)	4.0 (2.0 to 5.8)

**Table 5:** Inter-observer comparison of cut and space measurements (Wilcoxon signed-rank test).

Measure (mm)	Surgeon A (Mean ± SD)	(Median ± IQR A)	Surgeon B (Mean ± SD)	(Median ± IQR B)	*p*-Value (Wilcoxon Signed-Rank Test)	ICC	95% CI
Extension medial cut	13.73 ± 4.48	13.5 [11.00–17.00]	14.29 ± 4.09	14 [11.50–17.50]	0.61	0.99	0.98–0.99
Extension medial space	3.47 ± 2.43	3 [1.50–4.50]	2.92 ± 2.06	3 [1.00–5.00]	0.091	0.82	0.68–0.91
Extension lateral cut	14.08 ± 5.36	14.5 [9.50–19.50]	14.32 ± 4.8	15 [10.00–20.00]	0.622	0.95	0.90–0.97
Extension lateral space	3.66 ± 2.14	3.5 [2.00–5.00]	4.03 ± 2.39	3.75 [2.00–5.50]	0.17	0.89	0.80–0.94
Flexion medial cut	13.53 ± 4.09	13 [11.00–15.00]	14.03 ± 3.89	15 [11.50–18.50]	0.498	0.97	0.94–0.98
Flexion medial space	2.31 ± 2.01	2 [0.50–3.50]	1.99 ± 1.44	2 [1.50–2.50]	0.656	0.56	0.30–0.75
Flexion lateral cut	13.5 ± 3.64	15 [11.50–18.50]	13.72 ± 3.53	15.5 [11.50–19.50]	0.713	0.98	0.96–0.99
Flexion lateral space	3.42 ± 2.73	3 [1.00–5.00]	3.3 ± 2.23	3 [2.00–4.00]	0.934	0.72	0.53–0.85

## Data Availability

Data available on request from the authors.

## Abbreviations

The following abbreviations are used in this manuscript:

BMI	Body Mass Index
VAS	Visual Analogic Scale
SD	Standard Deviation
TKA	Total Knee Arthroplasty
OA	Osteoarthrosis
ROM	Range Of Motion
